# A Narrative Review of Regional vs. General Anesthesia in Cancer Surgery: Immune Pathways and Long-Term Oncologic Outcomes

**DOI:** 10.7759/cureus.111149

**Published:** 2026-06-19

**Authors:** Jad Kabbara, Fawaz Ali, Ayah Zureiqat, Ghania Khan, Samer Naffouje

**Affiliations:** 1 Anesthesiology, Lake Erie College of Osteopathic Medicine, Erie, USA; 2 Chemistry, Baldwin Wallace University, Berea, USA; 3 General Surgery, Civil Hospital Karachi, Dow University of Health Sciences, Karachi, PAK; 4 Sugerical Oncology, Cleveland Clinic, Cleveland, USA

**Keywords:** anesthetic technique, general anesthesia, immune repose, regional anesthesia, surgical stress response, transurethral resection of bladder tumors (turbt), tumor biology

## Abstract

The choice between general anesthesia (GA) and regional anesthesia (RA) for oncologic surgery has been widely debated because of the implications for perioperative safety, patient comfort, cancer recurrence, and long-term survival. RA may attenuate surgical stress and preserve immune function, whereas GA, particularly with volatile agents and opioids, may impair immune surveillance. Clinical findings, however, remain inconsistent. The most consistent signals of benefit have been observed in non-muscle-invasive bladder cancer undergoing transurethral resection of bladder tumors (TURBT), where observational cohorts and limited randomized data suggest reduced recurrence with neuraxial techniques. In contrast, studies in colorectal and lung cancer have shown no significant differences in recurrence or survival. Variability in tumor biology, perioperative care, and anesthetic exposure likely contributes to these discrepancies. This narrative review synthesizes randomized trials and large cohort studies to identify tumor types most likely to respond to anesthetic modulation and to highlight important evidence gaps. Overall, any oncologic effect of anesthetic technique appears modest and tumor-specific, warranting further adequately powered, disease-focused trials.

## Introduction and background

The choice between general anesthesia (GA) and regional anesthesia (RA) for oncologic surgery remains an ongoing area of discussion influenced by perioperative safety, technical feasibility, and patient comfort. In recent years, increasing attention has been directed toward whether anesthetic technique may also influence long-term cancer outcomes, including recurrence and survival. Early studies suggested that RA may reduce postoperative tumor recurrence and metastasis by attenuating the surgical stress response, preserving immune surveillance, and reducing perioperative neuroendocrine stress [1].

In contrast, exposure to volatile anesthetics and opioids during GA may contribute to perioperative immunosuppression and impairment of natural killer (NK) cell activity, an important component of antitumor immunity [2,3]. Regional anesthetic techniques have also been associated with reduced systemic inflammation, reflected by biomarkers such as the neutrophil-to-lymphocyte ratio (NLR) [2].

Despite these proposed biologic mechanisms, evidence regarding oncologic outcomes remains inconsistent. Previous systematic reviews and meta-analyses have been limited by variability in tumor type, disease stage, anesthetic protocols, and perioperative management strategies. Although several large studies have failed to demonstrate a consistent survival benefit associated with RA, smaller investigations continue to identify biologically relevant immune-modulating effects linked to anesthetic technique [2]. Tedore provided an early comprehensive overview of the mechanistic and clinical landscape, noting that while neuraxial, paravertebral, and local infiltration techniques each confer theoretical immunologic advantages over volatile-based GA, the clinical evidence at that time was largely retrospective and methodologically heterogeneous [4]. More recently, a comprehensive meta-analysis by Xie et al. pooling data across multiple tumor types suggested a reduction in recurrence and metastasis rates with RA [5], while the critical review by Nair et al. challenged the hypothesis, identifying significant methodological flaws in prior supporting studies [6]. Salib et al. reinforced a tumor-specific interpretation, noting that the strongest clinical benefit remains confined to bladder cancer [7]. Consequently, the overall impact of anesthetic approach on long-term oncologic outcomes remains uncertain. This narrative review aims to synthesize current evidence regarding the oncologic implications of RA versus GA while highlighting important limitations and areas for future research.

Methods

This was a narrative review comparing GA and RA with respect to oncologic outcomes. To identify relevant literature, two major electronic databases, PubMed and the Cochrane Library, were systematically searched between August and October 2025. A broad range of keywords was used, including “general anesthesia,” “regional anesthesia,” “volatile anesthetics,” and “epidurals,” as well as terms related to cancer outcomes such as “cancer recurrence,” “cancer progression,” “progression-free survival,” and specific malignancies including “prostate cancer,” “bladder cancer,” “ovarian cancer,” and “colorectal cancer.”

To identify eligible publications, strict inclusion criteria were applied. Included studies consisted of randomized controlled trials, prospective and retrospective cohort studies, case-control studies, systematic reviews, and meta-analyses involving adult or pediatric patients undergoing surgery for malignancy. Studies were excluded if they consisted of case reports, editorials, conference abstracts lacking peer-reviewed data, or animal-based experimental studies.

Three independent reviewers screened and evaluated all studies to ensure inclusion criteria were met and to minimize errors. Discrepancies were resolved through discussion until consensus was reached among all reviewers. Extracted data included study design, patient population, type of anesthetic exposure, and malignancy type.

Because this study was conducted as a narrative review, no formal statistical analysis or risk-of-bias assessment was performed. Instead, findings were synthesized descriptively to compare anesthetic approaches across different malignancies while highlighting key mechanisms, clinical evidence, and ongoing controversies within the field.

## Review

Pathophysiology

Understanding the pathophysiology and mechanisms underlying the potential suppression of cancer recurrence with RA compared to that with GA is critical, particularly in prostate cancer, because of the unique immunologic characteristics of its tumor microenvironment. Prostate cancer is typically considered immunologically ‘cold,’ with a suppressive tumor microenvironment, and androgen receptor signaling may contribute to immune modulation within the tumor milieu. Consequently, perioperative neuroendocrine and immune alterations may influence disease recurrence following prostatectomy [8]. The perioperative period represents a biologically vulnerable window during which circulating tumor cells (CTCs), micrometastases, and residual disease compete with the host immune system for survival. Anesthetic technique may influence this balance by altering neuroendocrine stress responses, innate and adaptive immunity, angiogenesis, and the tumor microenvironment.

Surgical stress activates the sympathetic nervous system and hypothalamic-pituitary-adrenal axis, resulting in surges of catecholamines, cortisol, and inflammatory mediators. This neuroendocrine cascade suppresses innate immunity, particularly NK cell cytotoxicity, which is essential for eliminating CTCs. NK cells recognize cancerous cells lacking normal major histocompatibility complex class I expression and induce apoptosis through granzyme and perforin release while secreting interferon-γ to inhibit angiogenesis. Consequently, perioperative stress may allow tumor cells released during surgery to evade immune clearance and metastasize [9]. RA minimizes this process by blocking afferent nociceptive input and reducing systemic opioid requirements, thereby attenuating hypothalamic-pituitary-adrenal and sympathetic activation. In contrast, volatile anesthetics and opioids used during GA may exacerbate immunosuppression, increasing the risk of perioperative tumor survival [10-12].

The choice of anesthetic technique also directly affects innate immunity, particularly NK cells and macrophages. Volatile anesthetics such as isoflurane and sevoflurane reduce macrophage phagocytosis, increase proangiogenic mediators such as vascular endothelial growth factor (VEGF), and impair NK cell function. Opioids may directly promote tumor growth through receptor-mediated pathways on cancer cells while simultaneously reducing NK cell cytotoxicity through μ-opioid receptor signaling on immune cells [12]. RA preserves NK cell activity and decreases circulating interleukin (IL)-6 and IL-8 levels by reducing opioid use and catecholamine surges. In breast cancer, which has been extensively studied, sevoflurane promotes VEGF release and postoperative tumor cell peaks, whereas propofol suppresses VEGF-C and appears less immunosuppressive, although short-term recurrence-free survival remains unaffected. RA combined with propofol, including paravertebral or pectoral nerve blocks, preserves NK cytotoxicity, lowers postoperative NLRs, and enhances interleukin-2 levels compared with sevoflurane-opioid anesthesia. These findings support a biologically favorable immune profile, although a consistent survival benefit has yet to be demonstrated [9].

Long-term tumor control depends on T-cell-mediated adaptive immunity, with CD4+ helper T cells organizing the immune environment and CD8+ cytotoxic T lymphocytes necessary for sustained tumor clearance. GA may shift the immune balance toward tumor tolerance by reducing T-cell proliferation and increasing regulatory T-cell activity, whereas RA maintains T-cell reactivity, potentially fostering a more favorable adaptive immune profile. The tumor microenvironment, including endothelial cells, fibroblasts, and infiltrating immune cells, further influences recurrence risk. Perioperative immunosuppression promotes angiogenesis and metastatic potential through increased VEGF signaling and matrix-remodeling enzymes, enhancing vascular permeability and facilitating CTC seeding. GA with volatile agents amplifies VEGF signaling, whereas RA appears to limit perioperative angiogenic drive, potentially reducing metastatic potential in susceptible tumor types [10].

Not all tumors respond equally to anesthetic modulation, and differences in tumor immunobiology help explain this variability. Bladder cancer, characterized by a high mutational burden and immunogenic profile, relies heavily on intact innate immune surveillance, which may explain why several studies suggest reduced recurrence with RA. Gynecologic cancers such as ovarian and fallopian tube carcinoma, which are highly vascular and angiogenesis-driven, may similarly benefit from RA through regulation of VEGF and inflammatory cytokines, as suggested by reports of extended progression-free survival. In contrast, the immune-cold microenvironment of prostate cancer, dominated by androgen receptor signaling, appears less sensitive to anesthetic modulation, with only modest influence on recurrence through neuroendocrine and opioid-sparing pathways. Colorectal cancer (CRC), characterized by low NK cell infiltration and strong stromal and microbiota influences, demonstrates limited survival benefit, potentially reflecting resistance to perioperative immune regulation. Breast cancer demonstrates moderate sensitivity; while propofol-based total intravenous anesthesia (TIVA) and regional techniques preserve NK function and reduce VEGF release, large trials have not confirmed a consistent survival benefit [11].

Overall, the pathophysiologic rationale linking anesthetic selection to cancer outcomes centers on tumor biology and perioperative immune regulation. GA, particularly with volatile agents and opioids, may compromise innate and adaptive immune defenses, whereas RA attenuates neuroendocrine stress, reduces angiogenic signaling, and preserves immune surveillance. Tumor heterogeneity, however, likely determines the clinical relevance of these effects. Immunologically active tumors such as bladder and gynecologic cancers may be more sensitive to perioperative anesthetic modulation, whereas immune-cold tumors such as prostate cancer and CRC appear less responsive. Understanding these mechanisms may ultimately support precision perioperative oncology strategies that integrate anesthetic selection with immunotherapy and individualized cancer care.

Evolution of cancer/inflammatory system 

The perioperative period has increasingly been recognized as a crucial determinant of cancer progression and recurrence. The physiologic stress of surgery activates the hypothalamic-pituitary-adrenal axis and the sympathetic nervous system, leading to the release of catecholamines, cortisol, and inflammatory mediators. This stress response collectively suppresses immune surveillance, with NK cells among the most vulnerable components of the innate immune system [2].

Cancer is characterized by uncontrolled cellular proliferation driven by genetic and epigenetic alterations that disrupt normal regulatory mechanisms of growth, differentiation, and apoptosis [13,14]. These changes enable tumor cells to evade immune destruction and invade surrounding tissues, ultimately promoting metastasis. While early stages such as metaplasia and dysplasia represent precancerous transformations, detailed discussion of their cellular mechanisms extends beyond the scope of this review. Instead, the focus of this review is on how the perioperative environment, including anesthetic technique, may influence immune surveillance and inflammatory responses that facilitate tumor progression and recurrence [15-18].

The interaction between perioperative factors and the tumor microenvironment is critical, as surgery-induced inflammation, angiogenesis, and immune modulation may enhance tumor growth and metastasis. Understanding how anesthetic agents influence these pathways may provide opportunities to improve long-term oncologic outcomes [18].

Although early retrospective studies suggested that RA might influence cancer outcomes, more recent high-quality evidence has provided limited support for this hypothesis. The impact of anesthetic technique on recurrence and survival appears subtle, potentially tumor-specific, and often clinically insignificant in larger controlled populations.

Evidence supporting a benefit of regional anesthesia

Several systematic reviews and meta-analyses have evaluated the effect of anesthetic technique on cancer outcomes, suggesting that the potential benefits of RA may vary according to tumor type.

Bladder Cancer

Bladder cancer provides some of the strongest evidence supporting a clinically meaningful influence of anesthetic choice. Tumor-specific analyses have demonstrated decreased recurrence rates in patients receiving RA for non-muscle-invasive disease [19,20]. A 2023 meta-analysis involving more than 13,000 bladder cancer patients reinforced these findings, reporting a 26% reduction in recurrence risk when RA was combined with GA during TURBT (p = 0.03) [21].

Prostate Cancer

A retrospective cohort study of patients with prostate cancer undergoing radical prostatectomy observed a 57% reduction in recurrence among patients receiving GA combined with epidural analgesia over a follow-up period of approximately 3-12 years (p = 0.012) [22]. Although promising, the nonrandomized design introduces selection bias and limits the strength of causal inference.

Gynecologic Cancers

For gynecologic malignancies, including ovarian, fallopian tube, and primary peritoneal cancers, patients managed with epidural analgesia in a study by Capmas et al. demonstrated higher rates of complete resection and longer progression-free survival (20.8 vs. 13.0 months, p = 0.021) and overall survival (62.4 vs. 41.9 months, p < 0.001) compared with patients receiving GA alone [23]. These findings suggest that reducing perioperative immune suppression and inflammation through RA may improve tumor control; however, prospective trials are needed to establish causality.

Breast Cancer

A randomized controlled trial demonstrated that, in breast cancer, a paravertebral block combined with propofol anesthesia lowered perioperative VEGF-C levels and altered inflammatory mediators compared with GA (p = 0.005) [24]. Although these biomarker changes suggest a more favorable tumor microenvironment, improvements in recurrence or survival have not been consistently demonstrated. This uncertainty was further highlighted by the large multicenter randomized controlled trial conducted by Sessler et al., which found no significant difference in breast cancer recurrence or survival between patients receiving RA with paravertebral block and propofol anesthesia versus volatile GA with opioid analgesia [23]. Given its randomized design and large sample size, this trial significantly influenced current understanding of the relationship between anesthetic technique and oncologic outcomes, emphasizing the inconsistency between mechanistic findings and clinical recurrence data.

Colorectal Cancer

Evidence in CRC remains mixed. A retrospective study of 749 patients undergoing surgery for CRC, including both colon and rectal cancers, reported improved five-year overall survival in patients receiving GA with epidural analgesia compared with GA alone (62% vs. 54%, p < 0.02) [25]. However, confounding variables and the nonrandomized study design limit definitive conclusions.

Overall, the potential oncologic benefit of RA appears strongest in bladder, prostate, and gynecologic cancers, whereas data for breast cancer and CRC remain inconclusive. Variability among studies likely reflects tumor-specific immune responses, differences in study design, and heterogeneity in perioperative management. Collectively, the available evidence supports the need for well-designed prospective studies to clarify how perioperative immune modulation influenced by anesthetic technique affects long-term cancer outcomes.

Evidence refuting the benefit of RA

Although early retrospective studies suggested that RA might influence cancer outcomes, more recent high-quality evidence has provided limited support for this hypothesis. The impact of anesthetic technique on recurrence and survival appears subtle, potentially tumor-specific, and often clinically insignificant in larger controlled populations.

Bladder Cancer

Although some analyses have suggested reduced recurrence with RA in non-muscle-invasive bladder cancer, larger cohort studies have demonstrated no significant difference in recurrence risk between spinal and general anesthesia [20]. These findings suggest that anesthetic technique may not meaningfully alter outcomes for certain procedures or disease stages.

Prostate Cancer

Evidence in prostate cancer remains inconclusive. While earlier retrospective studies indicated potential benefits associated with neuraxial anesthesia, more recent analyses controlling for tumor stage and pathology have demonstrated no consistent effect on recurrence-free survival [26]. Current evidence suggests that anesthetic choice alone is likely less influential than tumor biology and other perioperative factors affecting immune function.

Gynecologic Cancers

Studies evaluating epidural analgesia during cytoreductive surgery for ovarian cancer have not demonstrated statistically significant improvements in overall survival, and trends in disease-free survival remain inconclusive [23]. Limitations inherent to observational studies, including selection bias and unmeasured confounding, prevent definitive conclusions.

Breast and Lung Cancer

High-quality randomized controlled trials have challenged earlier hypotheses in both breast and lung cancer. In breast cancer, paravertebral blocks combined with propofol anesthesia showed no significant difference in recurrence or survival compared with volatile-based GA despite favorable biomarker changes [27]. Similarly, randomized trials in lung cancer have reported no oncologic benefit associated with adding epidural anesthesia to GA [28,29].

Colorectal Cancer

Evidence in CRC further supports these findings. Large population-based cohorts and systematic reviews have failed to demonstrate consistent benefits of RA on recurrence or survival [30,31]. Although anesthetic agents may modulate perioperative immune function, the clinical significance of these effects remains uncertain.

Overall, current evidence regarding the impact of anesthetic technique on cancer outcomes remains mixed and likely tumor-specific. While many high-quality trials and large cohort studies report no clear differences between RA and GA, limitations such as retrospective design, inadequate statistical power, and lack of stratification by tumor subtype may obscure modest but clinically meaningful effects. Future prospective studies stratified by tumor type and immune phenotype are needed to clarify whether certain patient populations may benefit from regional techniques.

Methodological critique

Nair et al. published a particularly pointed critical analysis challenging the foundational hypothesis that RA prevents cancer recurrence [6]. The authors identified several recurring methodological flaws in supportive studies, including inadequate control for confounders such as tumor stage and surgical technique, lack of blinding, heterogeneous definitions of recurrence, and absence of stratification by tumor immunophenotype. They concluded that no reliable causal link between anesthetic technique and cancer recurrence has been established and emphasized that the hypothesis, while biologically plausible, has not been validated in adequately designed prospective trials.

Table 1 summarizes the most clinically significant studies in this review, organized by cancer type and study design, including both supportive and refuting evidence.

**Table 1:** Summary of the most clinically significant studies included in this review GA: general anesthesia; RA: regional anesthesia; RCT: randomized controlled trial; NLR: neutrophil-to-lymphocyte ratio; PLR: platelet-to-lymphocyte ratio; NK: natural killer cell; VEGF: vascular endothelial growth factor; PFS: progression-free survival; OS: overall survival; NMIBC: non-muscle-invasive bladder cancer; TURBT: transurethral resection of bladder tumor; TIVA: total intravenous anesthesia; HPV: human papillomavirus; N/A: not applicable

Study (Author, Year)	Study Type	Cancer Type	Anesthetic Comparison	Key Finding	Direction of Effect	Notes / Limitations
Ní Eochagáin et al., 2018 [1]	RCT	Breast	RA vs. volatile GA	RA associated with lower NLR and PLR; improved return to intended oncological therapy	Favors RA (surrogate endpoints)	Single-center; biomarker/process endpoints only, not survival or recurrence
Aghamelu et al., 2021 [2]	RCT	Breast	Regional vs. volatile anesthesia	RA associated with lower serum NETosis expression and reduced recurrence risk signal	Favors RA (pilot signal)	Small pilot study; limited statistical power for clinical endpoints
Hayakawa et al., 2002 [3]	Animal in vitro study	N/A	IFN-γ / NK T-cell ligand	IFN-γ mediates inhibition of tumor angiogenesis via NK T-cell ligand α-galactosylceramide	Mechanistic background	Animal study; does not directly assess anesthetic technique
Grivennikov et al., 2010 [8]	Review	Multiple	N/A	Foundational framework linking inflammatory mediators and immune regulation to tumor promotion	Mechanistic background	Not a clinical anesthesia study; provides immunologic rationale
Kim et al., 2022 [9]	Narrative review	Breast	RA / propofol TIVA vs. volatile GA	Propofol-based TIVA and RA preserve NK cytotoxicity and reduce VEGF; no consistent survival benefit in large RCTs	Mixed	No meta-analytic pooling; narrative synthesis only
Murphy et al., 2023 [10]	Narrative review	Multiple	Various RA vs. GA techniques	RA may reduce perioperative immunosuppression; clinical translation to survival benefit remains unproven	Mixed / Inconclusive	Narrative design; highlights heterogeneity across studies
Vrbanović Mijatovicet al., 2022 [11]	Narrative review	Multiple	RA vs. GA	Summarizes mechanistic and clinical literature; no definitive clinical benefit confirmed for RA	Inconclusive	Narrative design; limited new primary data
Montejano and Jevtovic-Todorovic, 2021 [12]	Narrative review	Multiple	Volatile vs. IV anesthetic agents	Reviews dual role of anesthesia as potential friend or foe; mechanistic focus on immune and tumor biology	Mixed	Narrative design; mechanistic emphasis; no primary data
Tsui et al., 2010 [13]	Retrospective cohort study	Prostate	GA + epidural vs. GA alone	Epidural anesthesia associated with reduced cancer recurrence rates after radical prostatectomy	Favors RA	Retrospective; selection bias; limited confounding control
Hanahan, 2022 [14]	Review	Multiple	N/A	Updated hallmarks of cancer framework including tumor microenvironment, immune evasion, and epigenetic reprogramming	Mechanistic background	Not a clinical anesthesia study
Hanahan and Weinberg, 2000 [15]	Review	Multiple	N/A	Original hallmarks of cancer: seminal oncology framework for uncontrolled proliferation, invasion, and metastasis	Mechanistic background	Not a clinical anesthesia study
Giroux and Rustgi, 2017 [16]	Review	Multiple (epithelial)	N/A	Metaplasia as precursor to dysplasia-cancer sequence; tissue injury adaptation mechanisms	Mechanistic background	Not a clinical anesthesia study
Evans et al., 2016 [17]	Review	Barrett's esophagus	N/A	Evolving management of metaplasia and dysplasia in Barrett's epithelium	Mechanistic background	Not a clinical anesthesia study
Schiffman and Wentzensen, 2013 [18]	Review	Cervical	N/A	HPV infection and multistage carcinogenesis of cervical cancer; models perioperative carcinogenesis context	Mechanistic background	Not a clinical anesthesia study
Zhang et al., 2021 [19]	Systematic review & meta-analysis	Multiple (late-stage)	Regional vs. GA	RA associated with modestly reduced recurrence in late-stage cancer patients across pooled data	Favors RA (modest)	Heterogeneous tumor types; largely retrospective source studies
Wang et al., 2023 [20]	Systematic review & meta-analysis	Bladder (NMIBC)	Regional vs. GA	Mixed findings; some cohorts show no significant recurrence difference between spinal and GA for NMIBC	Mixed / No consistent benefit	Inconsistent findings across included studies; high heterogeneity
Illias et al., 2023 [21]	Meta-analysis	Bladder (NMIBC)	RA + GA vs. GA alone (TURBT)	26% reduction in recurrence risk with RA (p = 0.03); largest bladder-specific meta-analysis to date	Favors RA	Includes observational studies; residual confounding possible
Biki et al., 2008 [22]	Retrospective cohort	Prostate	GA + epidural vs. GA alone	57% reduction in biochemical recurrence with epidural over 3–12 yr follow-up (p = 0.012)	Favors RA	Retrospective; selection bias; landmark early study but non-randomized
Capmas et al., 2012 [23]	Retrospective cohort	Ovarian/ gynecologic	Epidural analgesia vs. GA alone	Longer PFS (20.8 vs. 13.0 mo, p = 0.021) and OS (62.4 vs. 41.9 mo, p < 0.001) with epidural	Favors RA	Retrospective; unmeasured confounding; single institution
Looney et al., 2010 [24]	RCT	Breast	Paravertebral block + propofol vs. volatile GA	RA lowered perioperative VEGF-C (p = 0.005); no difference in recurrence or survival demonstrated	Favors RA (biomarkers only)	Biomarker primary endpoint; underpowered for clinical oncologic outcomes
Holler et al., 2013 [25]	Retrospective cohort	Colorectal	GA + epidural vs. GA alone	Improved 5-yr OS with epidural (62% vs. 54%, p < 0.02)	Favors RA	Retrospective; confounding variables; mixed colon and rectal cancers
Tseng et al., 2014 [26]	Retrospective cohort study	Prostate	Spinal anesthesia vs. GA	No significant difference in recurrence-free survival between spinal and general anesthesia	No benefit of RA	Retrospective; one of earliest refuting prostate-specific studies
Sessler et al., 2019 [27]	Multicenter RCT	Breast	Paravertebral block + propofol vs. volatile GA + opioids	No significant difference in cancer recurrence or survival between groups	No benefit of RA	Landmark RCT; largest and most influential refuting trial in breast cancer
Xu et al., 2021 [28]	RCT	Lung	Epidural + GA vs. GA alone	No difference in recurrence-free or overall survival with epidural addition	No benefit of RA	Well-controlled RCT design; directly refutes lung cancer benefit hypothesis
Li et al., 2023 [29]	Systematic review & meta-analysis of RCTs	Multiple (lung, breast, colorectal)	Regional vs. GA	No significant improvement in long-term survival with RA across tumor types in RCT-only analysis	No benefit of RA	Restricted to RCTs; highest-quality evidence synthesis; most rigorous refuting review
Hasselager et al., 2022 [30]	Registry-based retrospective cohort	Colorectal	Epidural analgesia vs. no epidural	No significant improvement in colorectal cancer recurrence or OS with epidural analgesia	No benefit of RA	Large registry; national data; residual confounding possible
Subhadarshini and Taksande, 2024 [31]	Narrative review	Colorectal	Various RA vs. GA techniques	No consistent survival benefit identified; mechanistic rationale present but clinically unproven in colorectal cancer	Inconclusive	Narrative design; colorectal-specific focus
Xie et al., 2024 [5]	Meta-analysis	Multiple (mixed)	RA vs. GA	RA associated with significantly reduced recurrence and metastasis rates across pooled cancer types	Favors RA	Pooled heterogeneous tumor types; limits tumor-specific conclusions; further RCTs needed
Salib et al., 2025 [7]	Narrative review	Multiple (bladder, breast, colorectal, prostate)	RA vs. GA	Bladder cancer shows most consistent oncologic benefit; other cancers inconclusive; tumor-specific interpretation emphasized	Favors RA (bladder); Mixed (others)	Narrative design; no meta-analytic pooling; most recent comprehensive review
Tedore, 2015 [4]	Narrative review	Multiple	Neuraxial, paravertebral, local infiltration vs. volatile GA	Strong biological rationale for RA; clinical evidence at time of publication largely retrospective and inconsistent	Mixed / Inconclusive	Early foundational review; predates major RCTs (Sessler 2019, Xu 2021)
Nair et al., 2021 [6]	Critical methodological review	Multiple	RA vs. GA	Identified recurring methodological flaws in supportive studies; no reliable causal link between RA and cancer recurrence confirmed	Refutes RA benefit hypothesis	Highlights inadequate confounding control, lack of blinding, heterogeneous recurrence definitions in prior studies

## Conclusions

This review examined the influence of RA and GA on cancer outcomes, with a focus on immune modulation and tumor biology. While RA may preserve innate and adaptive immune function and reduce perioperative stress, clinical evidence remains inconsistent across malignancies. Some tumor types, such as bladder and certain gynecologic cancers, appear more responsive to anesthetic modulation, whereas others, including CRC and prostate cancer, show limited or no benefit. These differences likely reflect variation in tumor immunogenicity, tumor microenvironment, and reliance on angiogenesis.

Despite a strong biological rationale, current clinical data do not support a universal advantage of one anesthetic technique over another. Conflicting findings are influenced by heterogeneity in study design, patient populations, tumor characteristics, and anesthetic approaches. Ultimately, clarifying the role of anesthetic technique in cancer outcomes will require well-designed, adequately powered prospective randomized trials. At present, anesthetic selection should be guided primarily by patient safety and surgical considerations, while emerging evidence continues to inform its potential role in long-term oncologic outcomes.
